# Clustering of Unhealthy Behaviors: Protocol for a Multiple Behavior Analysis of Data From the Canadian Longitudinal Study on Aging

**DOI:** 10.2196/24887

**Published:** 2021-06-11

**Authors:** Zack van Allen, Simon L Bacon, Paquito Bernard, Heather Brown, Sophie Desroches, Monika Kastner, Kim Lavoie, Marta Marques, Nicola McCleary, Sharon Straus, Monica Taljaard, Kednapa Thavorn, Jennifer R Tomasone, Justin Presseau

**Affiliations:** 1 School of Psychology University of Ottawa Ottawa, ON Canada; 2 Clinical Epidemiology Program Ottawa Hospital Research Institute Ottawa, ON Canada; 3 Department of Health, Kinesiology & Applied Physiology Concordia University Montreal, QC Canada; 4 Montreal Behavioural Medicine Centre Le Centre intégré universitaire de santé et de services sociaux du Nord-de-l'Île-de-Montréal Montreal, QC Canada; 5 Department of Physical Activity Sciences University of Quebec in Montreal Montreal, QC Canada; 6 Research Center of the Montreal Mental Health University Institute Montreal, QC Canada; 7 Population Health Sciences Institute Faculty of Medical Sciences Newcastle University Newcastle upon Tyne United Kingdom; 8 Department of Food and Nutrition Sciences Laval University Quebec City, QC Canada; 9 Department of Medicine University of Toronto Toronto, ON Canada; 10 North York General Hospital Toronto, ON Canada; 11 Department of Psychology University of Quebec in Montreal Montreal, QC Canada; 12 ADAPT Science Foundation Ireland Research Centre Trinity College Dublin Dublin Ireland; 13 Comprehensive Health Research Centre NOVA Medical School Lisbon Portugal; 14 School of Kinesiology and Health Queen's University Kingston, ON Canada

**Keywords:** health behaviors, multiple behaviors, cluster analysis, network analysis, CLSA

## Abstract

**Background:**

Health behaviors such as physical inactivity, unhealthy eating, smoking tobacco, and alcohol use are leading risk factors for noncommunicable chronic diseases and play a central role in limiting health and life satisfaction. To date, however, health behaviors tend to be considered separately from one another, resulting in guidelines and interventions for healthy aging siloed by specific behaviors and often focused only on a given health behavior without considering the co-occurrence of family, social, work, and other behaviors of everyday life.

**Objective:**

The aim of this study is to understand how behaviors cluster and how such clusters are associated with physical and mental health, life satisfaction, and health care utilization may provide opportunities to leverage this co-occurrence to develop and evaluate interventions to promote multiple health behavior changes.

**Methods:**

Using cross-sectional baseline data from the Canadian Longitudinal Study on Aging, we will perform a predefined set of exploratory and hypothesis-generating analyses to examine the co-occurrence of health and everyday life behaviors. We will use agglomerative hierarchical cluster analysis to cluster individuals based on their behavioral tendencies. Multinomial logistic regression will then be used to model the relationships between clusters and demographic indicators, health care utilization, and general health and life satisfaction, and assess whether sex and age moderate these relationships. In addition, we will conduct network community detection analysis using the clique percolation algorithm to detect overlapping communities of behaviors based on the strength of relationships between variables.

**Results:**

Baseline data for the Canadian Longitudinal Study on Aging were collected from 51,338 participants aged between 45 and 85 years. Data were collected between 2010 and 2015. Secondary data analysis for this project was approved by the Ottawa Health Science Network Research Ethics Board (protocol ID #20190506-01H).

**Conclusions:**

This study will help to inform the development of interventions tailored to subpopulations of adults (eg, physically inactive smokers) defined by the multiple behaviors that describe their everyday life experiences.

**International Registered Report Identifier (IRRID):**

DERR1-10.2196/24887

## Introduction

Two-thirds of all annual deaths in Canada are caused by 4 noncommunicable chronic diseases: cancer, cardiovascular disease, diabetes, and chronic respiratory disease [1]. Approximately 12% of Canadians 65 years or older have lived with 2 or more of these chronic diseases, also known as multimorbidity [2]. Health behaviors such as physical inactivity, unhealthy eating, smoking tobacco, and alcohol use are leading risk factors for chronic diseases and play a central role in health status and quality of life [3]. The prevalence of risky health behaviors is high, with approximately 4 in 5 Canadian adults reporting at least one of these modifiable risk factors for noncommunicable chronic diseases [2].

Although the risk factors and consequences of multimorbidity have been studied extensively [4-6], much less attention has been paid to understanding how different combinations of multiple behaviors influence individuals’ life satisfaction and health. Our everyday lives are defined by multiple co-occurring health, social, family, personal, and work-related behaviors, each vying for the limited time, energy, and motivation available [7]. In spite of this, health behaviors tend to be studied and promoted largely separately from each other, resulting in guidelines and interventions for healthy aging siloed by specific behaviors. For example, Canada has distinct sets of guidelines for physical activity and sleep [8] and alcohol consumption [9]. In addition to focusing on single health behaviors, guidelines often do not consider the interconnectedness—or co-occurrence—of the various other family, social, work, hobby, and other behaviors that characterize daily life. Understanding how behaviors cluster and how such clusters are associated with physical and mental health, life satisfaction, and health care utilization may provide new opportunities to leverage this co-occurrence to promote multiple health behavior change interventions tailored to which behaviors co-occur for whom, thus better reflecting the real-world complexity of health care for aging Canadians.

International population data support the co-occurrence and clustering of health behaviors. For instance, Irish national data collected in the 2007 National Survey of Lifestyle, Attitudes, and Nutrition investigated the co-occurrence of smoking, alcohol use, physical inactivity, and unhealthy eating in adults aged 18 years and older [10]. In this cross-sectional analysis, the authors identified 6 clusters of behaviors to describe the population, which were labeled as (1) *healthy lifestyle* (characterized by people who had never smoked, high physical activity, highest healthy eating, and moderate alcohol use), (2) *former smokers* (former smokers who reported high physical activity, moderate alcohol use, and healthy eating), (3) *temperate* (moderately active and moderate drinkers who had never smoked), (4) *physically inactive* (people with low levels of physical activity, poor eating habits, and who reporting some smoking and high alcohol use), (5) *mixed lifestyle* (those who had never smoked and reported moderate physical activity and variable alcohol consumption), and (6) *multiple risk factor* (moderate physical activity, moderate to high alcohol use, and variable healthy eating). Although the cluster labels do not directly convey the co-occurrence of the behaviors, definitions of each label describe which behaviors co-occur most in each group in terms of the extent of the performance of each behavior. Beyond defining how behaviors cluster, they also showed that membership in each cluster was associated with demographic factors and health and well-being outcomes: those in the *healthy lifestyle* cluster were more likely to be women 65 years or older, be in the highest socioeconomic status (SES) group, and exhibit low psychological distress; those in the *former smokers* cluster were more likely than the healthy lifestyle cluster to be men and be in a lower SES group; the *temperate* cluster was associated with being male and being in a lower SES group as well as being younger; the *physically inactive* cluster was associated with being male aged 18 to 29 years and being in a lower SES group and having higher psychological distress; the *multiple risk factor* cluster had lowest reported energy and vitality, highest distress, lowest self-reported health, and lowest quality of life; and the *mixed lifestyle* cluster was associated with being male and younger and being in a lower SES group, with highest distress and lower energy and vitality. These findings show how the population can be segmented by the multiple health behaviors that characterize their lives and that these segmented clusters are socially patterned and associated with different health outcomes. It remains to be seen how such clusters may hold when focusing on adults aged 45 to 85 years and extending the behaviors under consideration beyond health behaviors. Identifying these clusters and their associations with demographic factors, general health, and health care utilization can inform the tailoring of future interventions targeting multiple behavior change.

In another study, Buck and Frosini [11] used data from the Health Survey for England to investigate the clustering of smoking, excessive alcohol use, unhealthy eating, and physical inactivity among adults aged 16 to 74 years between 2003 and 2008. They showed that in 2008, only 7% of people engaged in none of the behaviors and only 5% engaged in all 4; 63% of people engaged in 1 or 2, with the remaining 25% engaging in 3 or more behaviors. The most common co-occurring behaviors included either unhealthy eating and physical inactivity (more prevalent in women) or alcohol use, unhealthy eating, and inactivity (more prevalent in men). Men were more likely than women to have 3 co-occurring unhealthy behaviors, and those 65 years and older were more likely to have 2 co-occurring unhealthy behaviors than any other age group (16-24, 25-44, 45-64, and ≥65 years).

Although the importance of co-occurring health behaviors is clear, the role of nonhealth behaviors should not be overlooked. Using Australian population survey data collected in 2007 and 2009 in the Household Income and Labour Dynamics of Australia survey, we showed that other behaviors co-occurred with healthy eating and to some extent differed between men and women aged 18-65 years [12]. For Australian women, some family behaviors (caring for young children) and work behaviors (being employed in a managerial position) were associated with healthy eating, whereas not working was negatively associated with healthy eating. For men, the co-occurrence of work-related behaviors with healthy eating depended on the type of job category (positive for managers and negative for laborers). The findings highlight that nonhealth behaviors also co-occur with health behaviors and underscore the connectedness of the multiple behaviors in daily life.

The examples discussed so far have examined co-occurring health behaviors in the general population. However, some studies have focused on adults aged 50 years and older. For example, one study specifically focusing on older adults involved cross-sectional data collected in Germany in 2006, involving 982 men and 1020 women aged 50 to 70 years [13]. The authors investigated how 4 health behaviors (tobacco use, alcohol use, unhealthy eating, and physical inactivity) clustered in the population of adults in one German state and reliably grouped individuals by the unhealthy behaviors that they engaged in: 25% were defined by the lack of unhealthy behaviors, 21% as inactive, 18% as low fruit and vegetable eaters, 13% as smokers with other risk behaviors, and 23% as drinkers with other risk behaviors [13]. Although some adults were only described using one unhealthy behavior, over one-third had multiple unhealthy behaviors that clustered together. Furthermore, membership in each cluster was also patterned in terms of sociodemographic factors: those with multiple co-occurring unhealthy behaviors tended to be men, living alone, and of a lower SES. This study focused on a limited set of unhealthy behaviors, and it remains unclear which other behaviors characterizing daily life may also co-occur.

A different methodological approach was taken by Shaw and Agahi [14], who used baseline data from the 1998 Health and Retirement Study [15], which collected data from adults older than 50 years, to form *health behavior profiles*. In total, 12 health behavior profiles were constructed based on combinations of smokers versus nonsmokers, physically active versus inactive, and those who reported no versus moderate versus heavy alcohol consumption. Profiles varied widely in the percentage of participants captured in each profile. The 6 most prevalent profiles included the following: (1) *physically inactive, nondrinkers, who do not smoke* (6702/19,662, 34.1%); (2) *physically active, nondrinkers, who do not smoke* (4662/19,662, 23.7%); (3) *physically active, moderate drinkers, who do not smoke* (1986/19,662, 10.1%); (4) *physically inactive, moderate drinkers, who do not smoke* (1684/19,662, 8.6%); (5) *physically inactive, nondrinkers, who smoke* (1274/19,662, 6.5%); and (6) *physically active, nondrinkers, who smoke* (820/19,662, 4.2%).

Taken together, population survey data worldwide are converging on the idea that health behaviors cluster differently across the population and that the resulting clusters have distinct sociodemographic and health outcome patterns. However, to our knowledge, this has only been explored to a limited extent in Canadian data. Canadian National Population Survey data have shown that physical inactivity, alcohol use, and smoking co-occur [16,17]. Understanding how behaviors co-occur at a population level may provide novel ways of developing support and guidance for patients and clinicians and preventing noncommunicable chronic diseases in the general population using a multiple behavior approach.

To this end, we aim to leverage cross-sectional baseline data from the Canadian Longitudinal Study on Aging **(**CLSA) [18]. By 2031, 1 in 4 Canadians will be aged 65 years or older, and the CLSA aims to understand the determinants of health and wellness as people age [19]. For our purposes, we will use CLSA baseline data to address the following objectives: (1) describe how health behaviors cluster, (2) describe how other behaviors of everyday life (family, work, hobby, and community behaviors) cluster, (3) identify sociodemographic factors (sex, age group, marital status, income, country of birth, and social support availability) associated with cluster membership, (4) examine whether life satisfaction and health differ across clusters, (5) examine whether and which clusters of behavior are associated with health care utilization, and (6) examine whether clusters of health behaviors are associated with nonhealth behaviors.

## Methods

### Overview

The CLSA is a national longitudinal study designed to assess the biological, physical, societal, and psychosocial factors involved in healthy aging [18]. CLSA baseline data collection was conducted between 2010 and 2015 and involved 2 approaches: (1) a *tracking* cohort (n=21,241) that responded to questions administered by a 60-minute computer-assisted telephone interview and (2) a *comprehensive* cohort (n=30,097) that involved a 90-minute in-person interview and a data collection site visit. A 30-minute *maintaining contact questionnaire* was also administered by telephone to both cohorts 18 months after the initial contact to collect supplementary data from the same cohort (all of which form the baseline data collection used in our planned analysis).

### Participants

Participants were recruited through random-digit dialing, provincial health registries, and the Canadian Community Health Survey on Healthy Aging [19,20]. Exclusion criteria for the CLSA included residents living in 3 territories and First Nations reserves, full-time members of the Canadian Armed Forces, people living with cognitive impairments, and individuals living in institutions (including 24-hour nursing homes) [21]. This study included 51,338 French- and English-speaking Canadians (26,155/51,338, 50.95% female) aged between 45 and 85 years at the time of enrollment. The average participant age was 62.98 years (SD 10.43), with 26.15% (13,427/51,338) aged between 45 and 54 years, 31.98% (16,420/51,338) aged between 55 and 64 years, 23.37% (11,996/51,338) aged between 65 and 74 years, and 18.5% (9495/51,338) aged between 75 and 85 years. A full description of demographic characteristics of the sample as well as summary data across all measured variables is available in the CLSA baseline data report [19].

### Variable Selection

#### Selection Approach

Variable selection can be performed by *objective* or *subjective* approaches. Objective methods rely on data-driven techniques (eg, forward or backward selection) and techniques such as factor analysis and principal component analysis for dimension reduction to arrive at a parsimonious set of features for inclusion in a model [22]. In contrast, subjective approaches are generally driven by expert opinions and/or theory-driven research questions. Due to the manageable number of variables in the CLSA related to our research question, objective or data-driven approaches to feature selection were not required. Rather, based on our research objectives and the data collected by CLSA, we identified by group consensus an initial set of variables assessing health behaviors, nonhealth behaviors, sociodemographic indicators, general health and well-being, and health care service utilization. Our decisions were also shaped by issues of survey design (eg, skip questions), knowledge of basic summary statistics for baseline CLSA data [19], and our own supplementary summary statistics on the baseline data. Multimedia Appendix 1 provides a description of variables in each category (eg, health behaviors and nonhealth behaviors) to be used in the analyses, along with example items.

#### Health Behaviors

*Physical activity* and *sedentary behavior* were measured using the Physical Activity Scale for the Elderly [23], which assesses the frequency of sedentary behavior, walking, light physical activity, moderate physical activity, strenuous physical activity, and exercise. The items asked participants to report on their activity levels over the previous 7 days on a scale of 1 (never) to 4 (often, 5-7 days). A recent report published by Statistics Canada focusing on the relationship between physical activity and lung functioning [24] merged light and moderate physical activity together and merged strenuous physical activity and exercise together based on issues with question prompts and conceptual overlap between question items. To facilitate dimension reduction, we opted for a similar approach in which the Physical Activity Scale for the Elderly subscale items were merged to represent sitting, walking, light or moderate physical activity, and strenuous physical activity or exercise.

*Fruit and vegetable consumption* was assessed using one item from the Seniors in the Community Risk Evaluation for Eating and Nutrition questionnaire [25]. The item asks respondents how many servings of fruits and vegetables they eat in a day. The original scale was reverse coded such that higher scores indicate more fruit and vegetable consumption.

*Smoking behavior* was measured using a skip-question framework in the CLSA. Participants who answered “no” to the question “have you smoked at least 100 cigarettes in your life” and responded “yes” to the question “have you ever smoked a whole cigarette” were subsequently asked whether they smoke occasionally, daily, or not at all in the past 30 days. Next, only participants who reported smoking occasionally or daily were asked follow-up questions pertaining to the frequency and types of tobacco products used. A descriptive analysis of the last 2 items showed that only a small minority of respondents (4845/51,338, 9.44%) engaged in occasional or daily smoking, with the majority (30,558/51,338, 59.52%) not engaging in smoking behavior in the past 30 days. Although the frequency of smoking would be a more informative metric, any cluster analysis with smoking frequency would reduce the sample size to 4845 and would only represent people who have smoked within the past 30 days. In addition, we will assign a value of 0 to each respondent who responded “no” to the question “have you ever smoked a whole cigarette,” as these individuals also did not smoke in the past 30 days. A similar approach has been applied to skip structure data when missing data represent the absence of a behavior or psychological feature [26]. Ultimately, this creates 4 levels distinguishing between people who have never smoked and people who have smoked occasionally, daily, or not at all during a 30-day window.

*Alcohol use* was assessed with a single item asking participants how often they drank alcohol in the past 12 months on a scale from 1 (almost every day) to 7 (less than once a week). Responses will be reverse coded so that higher values indicate greater alcohol consumption.

Finally, *sleep* was also measured with a single item. Participants were asked how many hours of sleep they get, on average, during the past month and could respond with any value between 0 and 24.

#### Nonhealth Behaviors

Two items representing participation in *hobbies* were selected from the general health module. One item asked how much time participants spent playing board games, crossword puzzles, cards, sudoku, or jigsaw puzzles. The second question asked how much time participants spent singing in a choir or playing a musical instrument. Both items were originally scored on a scale from 1 (every day) to 5 (once a year or less); we will reverse code these variables so that higher values represent higher frequencies.

A social participation module was included in the CLSA baseline data collection that asked respondents about their tendencies to engage in various *community activities*, including church or religious activities; attending concerts, watching plays, or visiting museums; service club or fraternal organization activities; community or professional association activities; volunteer or charity work; participation in activities with family or friends outside the household; participation in sports or physical activities with others; participation in educational or cultural activities; and participation in other recreational activities. The CLSA contains 2 derived variables in the social participation module, one binary variable reflecting whether participants engaged in any social activities and another reflecting the frequency of participation in any activity over the past 12 months (0=no activities, 1=yearly, 2=monthly, 3=weekly, and 4=daily). We will use only the latter in our analysis.

The *caregiving* module contained questions pertaining to assisting others, how many others were assisted, the type of assistance, the people who the respondents help most often, and the personal and professional impacts of providing care to others. To reduce the number of items included in the analysis, we will use a derived variable in the CLSA data set, which indicates whether the respondent provided assistance to any person in the past 12 months (excluding aid rendered as part of a paid job or volunteer work). According to the descriptive analysis from CLSA baseline data [19], 44.4% (22,805/51,338) of participants reported aiding another person due to health conditions or other limitations. This variable will be recoded as 0 (did not provide assistance) or 1 (did provide assistance).

Finally, the CLSA participants were asked whether they used any *social networking sites* (eg, Facebook, LinkedIn, MySpace, MSNGroups, and Twitter). Our preliminary descriptive analysis showed that 44.7% (22,959/51,338) of the participants reported using social networking sites, whereas 38.0% (19,518/51,338) of the participants were not social networking site users, and 17.3% (8861/51,338) of responses were either missing or nonresponses. Although more detailed follow-up questions were subsequently posed to respondents, including these items would substantially reduce the sample size available for analysis. Given the skip-question structure of the CLSA social networking site module, we will include a binary variable representing the use (1) or nonuse (0) of social networking sites.

#### Sociodemographic Indicators

We will use several sociodemographic indicators in our analysis. These include age, as grouped in the CLSA data set (45-54, 55-64, 65-74, or 75-85 years); sex (male or female); country of birth (recoded as 0=Canada or 1=other); marital status (single, married or common-law, widowed, divorced, or separated); household income (five income levels); retirement status (completely retired, partly retired, or not retired); and working status (yes or no to the question “are you currently working at a job or business.” In addition, participants responded to 19 questions from the Medical Outcomes Study (MOS) Social Support Survey [27]. The MOS is scored according to 5 subscales: tangible social support, affection, positive social interaction, emotional support, and informational support. The MOS overall support index is also scored in the CLSA baseline data set. To reduce the number of constructs in our analyses, we will use the overall support index, scored from 0 (low support) to 100 (high support).

#### General Health and Life Satisfaction

Three single-item measures were selected from the CLSA’s general health module. These include an indicator of general health (“in general, would you say your health is excellent, very good, good, fair, or poor?”), mental health (“in general, would you say your mental health is excellent, very good, good, fair, or poor?”), and perceptions of healthy aging (“in terms of your own healthy aging, would you say it is excellent, very good, good, fair, or poor?”). Items were originally scored from 1 (excellent) to 5 (poor) but will be reverse coded. In addition, a composite score from the Satisfaction With Life Questionnaire [28] will be used in the analysis. The Satisfaction With Life Questionnaire is scored according to Deiner [29] on a scale ranging from 1 (extremely dissatisfied) to 7 (extremely satisfied). Finally, BMI will be used as an indicator of physical health.

#### Health Care Utilization

Three single-item questions were selected from the CLSA’s health care utilization module. These items represent emergency department visits (“Have you been seen in an Emergency Department during the past 12 months?”), hospital admittance (“Were you a patient in a hospital overnight during the past 12 months?”), and nursing home use (“Were you a patient in a nursing home or convalescent home during the past 12 months?”). All responses will be coded as yes (1) or no (0).

### Cluster Analysis Overview

We will use cluster analysis to cluster *individuals* based on their behaviors and network community detection algorithms to cluster *variables* based on the strength of conditionally independent pairwise relationships between variables.

Classifying data through the assignment of classes to objects in a data set is a common application of machine learning (ie, “set of methods that can automatically detect patterns in data, and then use the uncovered patterns to predict future data” [30]). Classification algorithms fall into 3 categories: supervised learning, semisupervised learning, and unsupervised learning. In supervised learning, the relationships between the input and target variables are known. An algorithm is *supervised* in that it can be trained on a data set that contains correct classifications. Data sets containing these correct classifications are referred to as *labeled data*, in contrast to *unlabeled data*, in which the correct classifications are not known. In semisupervised learning, a combination of labeled and unlabeled data is used to model the data, whereas in unsupervised learning, the model works on its own to discover patterns in unlabeled data [31].

Cluster analysis is a type of unsupervised machine learning that comprises a set of methods for identifying distinct characteristics in heterogeneous samples and clustering them into homogenous groups [32]. When the target number of clusters (*k*) is known, partitioning-based clustering arguments such as *k*-means, *k*-medoids, or model-based clustering approaches are appropriate. However, when *k* is unknown, as is the case with clusters of Canadians based on health and nonhealth behaviors, hierarchical clustering is a suitable method [32].

The hierarchical structure of the data can be obtained by clustering individual data points in a bottom-up approach (ie, agglomerative clustering) or by partitioning a single cluster into smaller clusters until each cluster is a single observation through a top-down approach (ie, divisive clustering). Divisive methods are rarely used in practice owing to their heavy computational requirements [33]. In agglomerative hierarchical clustering, each individual data point is initially treated as its own cluster. The methodological process is as follows [34]: each data point is assigned to its own cluster, the distance (ie, the similarity or dissimilarity between each cluster) between each cluster is calculated, the pair of clusters with the shortest distance between them is selected and merged into a single cluster, the distances between the new cluster and all other clusters are recalculated, and these steps are repeated until only one cluster remains. However, a single cluster (*k*=1) is unlikely to be informative; researchers can identify the number of clusters that best describe the data (eg, *k*=5) through subjective criteria and/or with the aid of statistical tests that have been developed for this purpose.

Several measures of *distance* are widely used in practice, although the Gower distance [35] is appropriate for mixed data (binary, ordinal, and continuous). In addition to selecting a measure of distance, hierarchical agglomerative clustering also requires a linkage method to be specified to define how the distance between clusters is calculated. Different methods exist for specifying the anchor points used to calculate the distance between clusters (ie, how the distances between clusters are *linked*). For example, *single or minimum linkage* calculates the minimum distance between data points in each cluster, whereas *centroid linkage* calculates the distance between the center of each cluster [31]. No consensus exists as to which linkage method is superior, although it is recognized that final clustering solutions may differ based on the linkage method selected [33].

### Network Analysis and Community Detection Overview

Another method for clustering data is through network community detection. In contrast to cluster analysis, which clusters *people* based on similarity or dissimilarity of selected features (eg, health behaviors), network community detection identifies groups of *highly connected variables* based on the strength of the connections. Before conducting the community detection analysis, a network must first be estimated. Networks consist of nodes and edges. In psychological networks, nodes represent psychological attributes (eg, emotions, behaviors, and symptoms) and edges represent relationships between nodes (eg, partial correlations) and can indicate the presence of a positive or negative relationship and the direction of the effect. Psychological networks are commonly estimated using a pairwise Markov random field (PMRF) [36,37]. In a PMRF, edges represent the conditional independence between a pair of connected variables. For cross-sectional data, the underlying models for the PMRF vary depending on the type of data. For example, a Gaussian graphical model is appropriate for multivariate normal continuous data, whereas Ising models are used for binary data, and mixed graphical models (MGMs) [38] have been developed for mixed data. Due to the large number of parameters often estimated in a PMRF, researchers often compute a regularized network by applying, for example, the least absolute shrinkage and selection operator [39] to produce a sparse or conservative network that reduces weak connections between nodes to 0, resulting in a more interpretable network. However, recent work has questioned whether regularization is required for low-dimensional data with large sample sizes [40].

Once a network has been estimated, an additional analysis can be conducted. For example, node centrality, a family of measures meant to indicate the importance of nodes within a network [37], can be computed. Other analytical options include calculating the explained variance and performing network comparison tests. In the cross-sectional data, a network is estimated by regressing all nodes onto each other. This enables the explained variance for each node to be calculated and visualized within the network itself [38]. Network comparison tests are permutation-based hypothesis tests that allow comparisons between 2 networks (eg, health behaviors for those aged 45-54 years and 55-64 years) based on their global structure, global strength (a measure of centrality), and differences between individual edges [41].

Finally, community detection algorithms can be applied to the network. Commonly used community detection methods in psychology include the spinglass algorithm [42], the walktrap algorithm [43], leading eigenvector [44], exploratory graph analysis [45], and the clique percolation algorithm [46,47]. When compared with alternative methods, the clique percolation algorithm addresses a central challenge in identifying communities within a network, namely, the ability to assign a node to multiple communities. Overlapping communities enable the identification of *bridge nodes* (nodes that connect 2 otherwise distinct clusters) and are therefore important for hypothesis generation [48]. Cliques are fully connected networks. The number of nodes (*k*) that must be connected can vary, with the smallest clique being *k*=3 (a closed triangle). When more than one set of cliques are adjacent in a network, they are said to form a community. In psychological networks, where edges are weighted (eg, representing correlations), only edges that surpass a certain threshold (*I*) are considered when identifying cliques [46,47,49].

### Proposed Analysis

#### Survey Weights

The CLSA baseline data set contains sample weights (to ensure representativeness of the sample), inflation weights (to improve the precision of estimates), and analytic weights (to estimate the relationships among variables) [50]. The CLSA recommends the use of inflation weights for the estimation of descriptive parameters and analytic weights for exploring the relationships among variables at a national or provincial level [50]. However, the statistical packages we have selected in our planned cluster analysis method do not have the option to include survey weights and will, therefore, not be applied. Given the relative novelty of network analysis methods in psychology, the appropriate role of survey weights in network methodology is unknown. To our knowledge, the only study that addresses this issue is the study by Lin et al [51], who opted not to include survey weights due to “a lack of established methods to do so for network models.” Therefore, weights will not be applied for the network analysis.

#### Preprocessing and Descriptive Statistics

All analyses will be conducted in R (R Core Team) [52]. Selected variables from the tracking (TRM) and comprehensive (COM) data sets will be merged and coalesced (eg, ALC_FREQ_TRM and ALC_FREW_COM coalesced into ALC_FREQ) using the tidyverse package [53]. Raw data will be visualized to inspect the univariate outliers. Means and SDs for all variables will be computed for continuous data, and frequencies and percentages will be computed for categorical data, overall and by sex and age group. Missing data will be handled with listwise deletion.

#### Cluster Analysis

##### Step 1

Variables will be reverse coded, when necessary, such that higher scores indicate greater frequency. Subsequently, all continuous and ordinal variables will be mean centered using the scale function in base R [52].

##### Step 2

We will run cluster analyses on the overall CLSA sample (all age groups) using 5 linkage methods (eg, complete linkage, single linkage, average linkage, centroid linkage, and Ward method). The research team will decide which method produces the most interpretable clustering solution; the selected linkage method will then be applied to all subset analyses grouped by age.

##### Step 3

Cluster analysis will be performed on health behavior variables for 5 groups (overall sample, 4 age groups) using the base R *hclust* function supported by the package *fastcluster* [54] to optimize performance. Gower distance will be computed using the *daisy* function in the *cluster* package [55].

##### Step 4

Once agglomerative cluster analysis has been performed, the next step is to determine the most interpretable number of clusters to represent the data. The cluster analysis literature has produced several competing methods to accomplish this task. To navigate these analytical options, we will use the NbClust package [56]. This approach allows for a consensus approach to determine the number of clusters, which best fits the data by computing 30 different indices and reporting the level of agreement between them. The output of this analysis is a list showing the agreement between statistical tests for various clustering solutions (eg, “10 indices propose 2 as the best number of clusters” and “7 indices propose 3 as the best number of clusters”). To allow for the synthesis of data-driven and expert consensus approaches, the research team will select the most interpretable clustering solution via a majority vote from the top 3 solutions identified via NbClust. In instances of an equal number of indices recommending the same cluster solution and/or disagreement within the research team, we will follow the advice of the NcClust package authors who recommend considering indices, which have performed the best in a seminal simulation study [57].

##### Step 5

When the optimal clustering solution has been identified, the clusters can be characterized by the number of individuals assigned to each cluster and by mean scores on each health-related variable via one-way analysis of variance tests to determine whether the mean levels of the health behaviors vary by cluster.

##### Step 6

Next, multinomial logistic regression will be used to determine whether clusters are associated with sociodemographic variables, indicators of physical and mental health (eg, life satisfaction), nonhealth behaviors, and health care utilization. Multinomial logistic regression is similar to logistic regression but is appropriate when the dependent variable has more than 2 levels (which will likely be the case for the number of identified clusters). The dependent variable will be the clusters we identify in the cluster analysis. To perform the analysis, we will use the *multinom* function from the *nnet* package [58].

#### Network Analysis

##### Step 1

Networks of health behaviors will be estimated using an MGM using the MGM package [38]. To the best of our knowledge, an MGM is the only available method for estimating a psychological network with mixed data. In the mixed model, the edges between nodes represent pairwise interactions and can be interpreted as the strength of conditional dependence [38]. A total of 5 networks will be estimated (all age groups, 45-54, 55-64, 65-74, and 75-85 years). In the mixed model, we will specify *lambdaSel=EBIC* to use the Extended Bayesian Information Criteria [59] for selecting the tuning parameter controlling regularization. The hyperparameter γ in the Extended Bayesian Information Criteria will be set to the default *lambdaGam=0.25,* and only pairwise interactions will be included in the model through setting *k*=2.

##### Step 2

For each network from step 2, networks will be visualized with qgraph [60] using the *averageLayout* function to compute a joint layout across networks.

##### Step 3

The NetworkComparisonTest package [41] will be used to conduct permutation-based hypothesis tests to determine whether networks differ from one another based on sociodemographic variables. We will specify *it=1000* to run 1000 iterations or permutations and will plot the results from the network structure invariance test.

##### Step 4

Next, community detection analysis will be performed on each network estimated in step 1 using the CliquePercolation package [49]. Although multiple options are available, we will detect overlapping communities by optimizing *k* and *I,* where *k* represents the minimum clique size and *I* represents the strength of the relationships between nodes required for classification as a community. Following the study by Blanken et al [61], we will determine the optimal threshold *I* for the fixed values of *k*=3-6 and will set the clique percolation algorithm to search through ranges of *I* from 0.01 to the largest edge weight in each network through increments of 0.001. We will choose the value of *k* that allows for the broadest community structure, and *I* will be selected based on the largest chi-square value for intensities with a ratio threshold over 2. The resulting networks will be visualized with colored nodes indicating community structures.

##### Steps 5 to 8

The processes described in steps 1 to 4 will be repeated for nonhealth behaviors and health behaviors in the same model. A summary of the proposed analysis and its connections with research questions is presented in Table 1. Further graphical representations of the analytical steps are provided in Multimedia Appendices 2 and 3.

**Table 1:** Overview of research questions and planned analysis.

Research questions	Planned analyses				
	Cluster analysis (analysis 1)	Multinomial logistic regression (analysis 1)	Network analysis (analysis 2)	Network community detection (analysis 2)	Network comparison tests (analysis 2)
Describe how health behaviors cluster in Canadians aged 45-85 years	✓	—	✓	✓	—
Describe how nonhealth behaviors cluster in Canadians aged 45-85 years	—	—	✓	✓	—
Identify sociodemographic factors associated with cluster membership; identify sociodemographic factors associated with network structures	—	✓	—	—	✓
Examine whether cluster are associated with health indicators	—	✓	—	—	—
Examine whether clusters are associated with health care utilization	—	✓	—	—	—
Examine whether clusters are associated with nonhealth behaviors	—	✓	—	—	—

### Data Availability

Data are available from the CLSA for researchers who meet the criteria for access to deidentified CLSA data. We will make R scripts public so that any researcher who independently gains access to data can reproduce the results. In addition, we will publish R Markdown documents so that the code and outputs of analysis can be viewed publicly on the Open Science Framework. Deviations from the protocol plan will be noted in the final report. Any additional analysis, not specified here, will be labeled as such and published in web-based supplemental materials.

## Results

Baseline data for the CLSA were collected from 51,338 participants aged between 45 and 85 years. Data were collected between 2010 and 2015. Secondary data analysis for this project was approved by the Ottawa Health Science Network Research Ethics Board (protocol ID #20190506-01H).

## Discussion

The scope, size, and rigor of the CLSA data set will provide us with an unprecedented opportunity to investigate how behaviors cluster. The findings will allow us to assess alignment with findings from other countries, while extending findings in novel ways by investigating how social, family, and work behaviors cluster alongside health behaviors typically investigated. Perhaps most importantly, this study will help to inform the development of novel health behavior change interventions tailored to subpopulations of adults defined by the behaviors that cluster within them. If behaviors co-occur, intervening on one may impact—or be impacted by—the others [62]. Interventions that target only one behavior may thus be undermined by the impact of a conflicting co-occurring behavior or miss an opportunity to leverage the enabling nature of a positively co-occurring behavior. In addition, targeting multiple co-occurring behaviors simultaneously has potential practical benefits, such as reduced expenses for intervention providers and reduced time commitments for those receiving the intervention. Understanding which behaviors co-occur and for whom is an important first step toward developing health behavior change interventions that address people’s actual challenges. In the context of a behavioral intervention development and testing framework [63], the proposed analysis will inform the foundational *basic behavioral science*—regarding patterns of co-occurring risky health behaviors and their associated outcomes—which precedes early phase behavioral trials.

This study will ultimately help to develop and evaluate more targeted interventions to support healthy aging and well-being in adulthood by identifying how clusters of co-occurring health behaviors are associated with sociodemographic factors, general health, and health care utilization. However, the proposed analyses are not without limitations. For example, many of the items selected for planned analysis are self-report, which have inherent strengths and weaknesses [64]. In addition, there are several points in the proposed analysis that require subjective decision making on behalf of the research team (eg, selecting variables for inclusion in models, data preprocessing decisions, selecting missing data procedures, and interpreting and selecting clustering solutions). We have sought to document these here to provide a clear explanation of decision processes, while acknowledging where and how researcher degrees of freedom are used to ensure transparency. Finally, we recognize that the analyses proposed are, to some extent, limited by their cross-sectional nature. Nevertheless, given the longitudinal nature of CLSA and planned future data releases, the proposed analyses have a number of novel implications and set the stage for planned future longitudinal analyses that extend the research questions to investigate changes in behavior clusters as a function of time both between and within individuals. Thus, this proposed study will establish the foundation for future analyses. More broadly, the methodological approaches proposed for this analysis lend themselves to replication in other similar data sets internationally, and we hope that the sharing of R code will help to enable this.

Finally, we emphasize that our choices of analytic methods are hypothesis generating, not hypothesis testing. However, if replicated, such findings may ultimately find their way into clinical practice guidance and public health guidance.

## Appendix

Multimedia Appendix 1 Variables to be included in the analysis.

Multimedia Appendix 2 Graphical representation of the cluster analysis and multinomial logistic regression analytical process.

Multimedia Appendix 3 Graphical representation of the network analysis and community detection process.

Multimedia Appendix 4 Peer-review report by the Canadian Institutes of Health Research.

## Abbreviations

ADAPT: Artificial Intelligence–Driven Digital Content Technology
CIHR: Canadian Institutes of Health Research
CLSA: Canadian Longitudinal Study on Aging
FRQS: Fonds de Recherche du Québec–Santé
MGM: mixed graphical model
MOS: Medical Outcomes Study
PMRF: pairwise Markov random field
SES: socioeconomic status
