# Supplementary material for: Clustering of Unhealthy Behaviors: Protocol for a Multiple Behavior Analysis of Data From the Canadian Longitudinal Study on Aging
Source: JMIR Res Protoc. 2021 Jun 11;10(6):e24887. doi: 10.2196/24887 (PMC8235290; doi:10.2196/24887)
Supplement: Multimedia Appendix 1 [file resprot_v10i6e24887_app1.docx]

**Multimedia Appendix 1**

| Table 1. Variables to be included in analysis | | |
| --- | --- | --- |
| **Behaviour** | **CLSA Variable** | **Item Question & Response Scale** |
| **Health Behaviours** | | |
| 1. **Sedentary Behaviour** | PA2_SIT | "Over the past 7 days, how often did you participate in sitting activities such as reading, watching TV, computer activities or doing handicrafts?" (*1 (never) to 4 (often, 5 to 7 days)*) |
| 1. **Walking** | PA2_WALK | "Over the past 7 days, how often did you take a walk outside your home or yard for any reason?" (*1 (never) to 4 (often, 5 to 7 days)*) |
| 1. **Light / Moderate Physical Activity** | Combined: PA2_MSPRT, PA2_LSPRT | "Over the past 7 days, how often did you engage in moderate sports or recreational activities such as ballroom dancing, hunting, skating, golf without a cart, softball or other similar activities?" (*1 (never) to 4 (often, 5 to 7 days)*)  Over the past 7 days, how often did you engage in light sports or recreational activities such as bowling, golf with a cart, shuffleboard, badminton, fishing or other similar activities? (*1 (never) to 4 (often, 5 to 7 days)*) |
| 1. **Strenuous Physical Activity / Exercise** | Combined PA2_SSPRT, PA2_EXER | "Over the past 7 days, how often did you engage in strenuous sports or recreational activities such as jogging, swimming, snowshoeing, cycling, aerobics, skiing, or other similar activities?" (*1 (never) to 4 (often, 5 to 7 days)*) |
|  |  | "Over the past 7 days, how often did you do any exercises specifically to increase muscle strength and endurance, such as lifting weights or push-ups, etc.?" (*1 (never) to 4 (often, 5 to 7 days)*) |
| 1. **Fruit and Vegetable Consumption** | NUR_FRTVEG | "In general, how many servings of fruits and vegetables do you eat in a day?" (*1 (seven or more) to 7 (less than two)*) |
| 1. **Smoking** | SMK_CURRCG | "At the present time, do you smoke cigarettes daily, occasionally or not at all?" (*1 (daily) 2 (occasionally) 0 (not at all))* |
| 1. **Alcohol Use** | ALC_FREQ | "About how often during the past 12 months did you drink alcohol?" (*1 (almost every day) to 7 (less than once a month)*) |
| 1. **Sleep** | SLE_HOUR_NB | "During the past month, on average, how many hours of actual sleep did you get at night?" (*continuous*) |
| **Non-Health Behaviours** | | |
| 1. **Caregiving for Family Member** | CAG_FPAS | This flag variable indicates whether the respondent provided assistance in the past 12 months to another person because of a health condition or limitation. Assistance provided as part of a volunteer organization or paid job is excluded. *(1= did not provide any assistance, 2 = did not provide any assistance or only provided financial assistance)* |
| 1. **Puzzles/crosswords** | GEN_BRD | "Playing board games, cards, crossword puzzles, jigsaw puzzles, or sudoku" (*1 (every day); 2 (several times a week); 3 (several times a month); 4 (several times a year); 5 (once a year or less)*) |
| 1. **Music** | GEN_MUSC | "Playing a musical instrument or singing in a choir." (*1 (every day); 2 (several times a week); 3 (several times a month); 4 (several times a year); 5 (once a year or less)*) |
| 1. **Community Activities** | SPA_DFRE | This variable categorizes respondents by the frequency of their participation in any type of community-related activity during the past 12 months. Activities include: 1) church or religious activities, 2) attending concerts, plays, or visiting museums, 3) service club or fraternal organization activities, 4) community or professional association activities, 5) volunteer or charity work, 6) participation in family/ friends activities out of household, 7) participation in sports or physical activities with others, 8) participation in educational or cultural activities, and 9) participation in other recreational activities. Scoring: 0 = no activities, 1 = yearly, 2 = monthly, 3= weekly, 4 = daily. |
| 1. **Social Media** | INT_SCLNTWRK | "Do you currently use social networking sites on the Internet? Examples of such sites include Facebook, LinkedIn, MySpace, MSNGroups, or Twitter." (*1 (yes), 2 (no); if yes, code use highest frequency of indicators*) |
| **Sociodemographic Factors** | | |
| 1. **Age** | AGE_DOB | "For some of the questions I’ll be asking, I need to know your exact date of birth" and "What is your age?" (*grouped: 45-54; 55-64; 65-74; 75-85*) |
| 1. **Sex** | SEX_ASK | M (1) / F (0) |
| 1. **Marital status** | SDC_MRTL | "What is your current marital/partner status?" *(Single/Married or common-law/Widowed/Divorced/Separated*) |
| 1. **Household income** | INC_TOT | "What is your best estimate of the total household income received by all household members, from all sources, before taxes and deductions, in the past 12 months?" (*<$20k/$20-$49k/$50-$99k/$100-$149k/$150k+*) |
| 1. **Social support availability** | SSA_DPALL | This derived variable measures the overall level of functional social support that is available to the respondent. It includes all aspects asked about in the MOS Social Support Survey. Higher scores indicate higher levels of functional social support (min = 0, max = 100) |
| 1. **Retirement status** | RET_RTRD | "At this time, do you consider yourself to be completely retired, partly retired or not retired?" (*1 (completely retired), 2 (partly retired), 3 (not retired)*) |
| 1. **Working** | LBF_CURR | "Are you currently working at a job or business?" (*1 (yes), 2 (no)*) |
| **Health and Life Satisfaction Outcomes** | | |
| 1. **General health** | GEN_DHDI | This derived variable indicates the respondent's health status based on his/her own judgement. It is a recoded version of the questionnaire responses so that higher scores now indicate a more positively perceived health status. (1 – poor, 5 – excellent) |
| 1. **Mental health** | GEN_DMHI | This derived variable indicates the respondent's mental health status based on his/her own judgement. It is a recoded version of the questionnaire responses so that higher scores now indicate a more positively perceived mental health status. (1 – poor, 5 – excellent) |
| 1. **Healthy aging** | GEN_OWNAG | "In terms of your own healthy aging, would you say it is excellent, very good, good, fair, or poor?" (1 - Excellent to 5 - Poor) |
| 1. **Life satisfaction** | SLS_DSCR | This variable describes participants’ satisfaction with life and is an aggregate score of the responses to the five items of the SWLS. Individual responses to each item in the SWLS range from 1 – strongly disagree to 7 – strongly agree, and this score is a sum of those responses. Higher scores indicate a greater satisfaction with life. (min 5, max 35) |
| 1. **BMI** | HWT_DBMI | Body Mass Index |
| **Health Care Utilization** | | |
| 1. **Emergency Department** | HCU_EMEREG | "Have you been seen in an Emergency Department during the past 12 months?" (*yes/no*) |
| 1. **Admitted to Hospital** | HCU_HLOVRNT | "Were you a patient in a hospital overnight during the past 12 months?" (*yes/no*) |
| 1. **Nursing Home** | HCU_NRSHM | "Were you a patient in a nursing home or convalescent home during the past 12 months?" (*yes/no*) |
