# Supplementary material for: Clustering of Unhealthy Behaviors: Protocol for a Multiple Behavior Analysis of Data From the Canadian Longitudinal Study on Aging
Source: JMIR Res Protoc. 2021 Jun 11;10(6):e24887. doi: 10.2196/24887 (PMC8235290; doi:10.2196/24887)
Supplement: Multimedia Appendix 2 [file resprot_v10i6e24887_app2.docx]

**Multimedia Appendix 2**

Graphical representation of the cluster analysis / multinomial logistic regression analytical process. Dendrogram image from simulated data of CLSA variables used to test analysis workflow. Values in tables were selected at random for illustration purposes. Variables labels (and full names): Walk (walking frequency), Fruit (fruit/vegetable consumption), Exer (exercise), Smoke (smoking frequency), L_PA (light physical activity), Sed (sedentary behaviour).


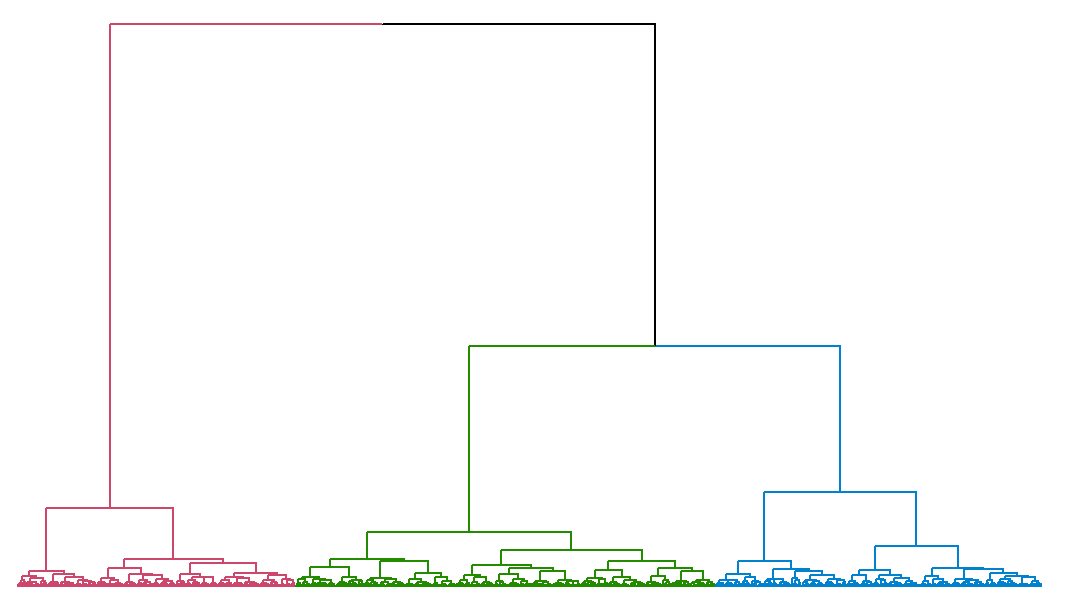

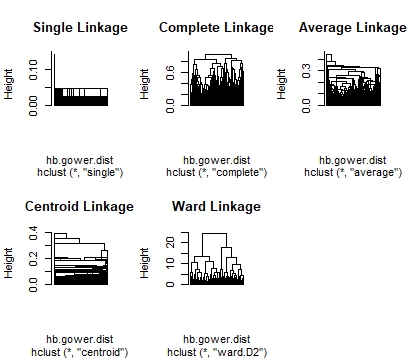


**Step 4:** determine optimal number of clusters (not shown) and link cluster membership to cases (3 cluster solution selected as an example)

**Step 1:** (not shown) data pre-processing; **Step 2:** run cluster analysis with 5 linkage methods. Decide on best method.

**Step 3:** Perform cluster analysis with linkage method selected in step 2.

| **Table x**. Standardized Means by Cluster | | | | | | | |
| --- | --- | --- | --- | --- | --- | --- | --- |
|  | | | | | | | |
| Cluster | Sed | Walk | Fruit | Exer | Smoke | L_PA | Alc |
| 1 | 0.85 | 0.97 | -0.06 | -0.75 | 0.76 | 0.15 | 1.02 |
| 2 | 0.05 | -0.07 | -0.79 | 0.07 | 0.10 | -.20 | -.22 |
| 3 | -0.72 | -0.75 | 0.96 | 0.80 | -0.75 | 0.01 | .15 |

3

2

1

**Step 6:** Multinomial logistic regression with clusters predicted by sociodemographic, health, healthcare utilization, and non-health bheaviour variables.

| Dataset with Clusters Assigned to Cases and Standardized Behaviours | | | | | | | | | | | | |
| --- | --- | --- | --- | --- | --- | --- | --- | --- | --- | --- | --- | --- |
| ID | Cluster | Walk | Fruit | | | | | Exer | Smoke | L_PA | Alc | Sed |
| 101 | 1 | 0.85 | 0.97 | | | | | -0.06 | -0.75 | 0.76 | 0.15 | 1.02 |
| 102 | 2 | 0.05 | -0.07 | | | | | -0.79 | 0.07 | 0.10 | -.20 | -.22 |
| 103 | 3 | -0.72 | -0.75 | | | | | 0.96 | 0.80 | -0.75 | 0.01 | .15 |
| 104 | 2 | -1.51 | -1.67 | | | | | 1.05 | 1.59 | -1.47 | 1.02 | 2.37 |
| 105 | 2 | -2.29 | -2.52 | | | | | 1.56 | 2.37 | -2.23 | -.22 | 3.14 |
| 106 | 3 | -3.07 | -3.38 | | | | | 2.06 | 3.14 | -2.98 | .15 | 3.92 |
| 107 | 1 | -3.86 | -4.24 | | | | | 2.57 | 3.92 | -3.74 | 1.59 | 0.85 |
| 108 | 1 | -4.64 | -5.10 | | | | | 3.08 | 4.69 | -4.49 | 2.37 | 0.05 |
| 109 | 2 | -5.42 | -5.96 | | | | | 3.59 | 5.47 | -5.25 | 3.14 | -0.72 |
| 110 | 3 | -6.21 | -6.81 | | | | | 4.09 | 6.24 | -6.00 | 0.97 | -1.51 |
|  | .  .  . | .  .  . | .  .  . | .  .  . | .  .  . | .  .  . | .  .  . | .  .  . |  |  |  |  |

| Dataset with Clusters Assigned to Cases and Standardized Behaviours | | | | | | | | |
| --- | --- | --- | --- | --- | --- | --- | --- | --- |
| ID | Cluster | Walk | Fruit | Exer | Smoke | L_PA | Alc | Sed |
| 101 | 1 | 0.85 | 0.97 | -0.06 | -0.75 | 0.76 | 0.15 | 1.02 |
| 102 | 2 | 0.05 | -0.07 | -0.79 | 0.07 | 0.10 | -.20 | -.22 |
| 103 | 3 | -0.72 | -0.75 | 0.96 | 0.80 | -0.75 | 0.01 | .15 |
| 104 | 2 | -1.51 | -1.67 | 1.05 | 1.59 | -1.47 | 1.02 | 2.37 |
| 105 | 2 | -2.29 | -2.52 | 1.56 | 2.37 | -2.23 | -.22 | 3.14 |
| 106 | 3 | -3.07 | -3.38 | 2.06 | 3.14 | -2.98 | .15 | 3.92 |
| 107 | 1 | -3.86 | -4.24 | 2.57 | 3.92 | -3.74 | 1.59 | 0.85 |
| 108 | 1 | -4.64 | -5.10 | 3.08 | 4.69 | -4.49 | 2.37 | 0.05 |
| 109 | 2 | -5.42 | -5.96 | 3.59 | 5.47 | -5.25 | 3.14 | -0.72 |
| 110 | 3 | -6.21 | -6.81 | 4.09 | 6.24 | -6.00 | 0.97 | -1.51 |
|  | .  .  . | .  .  . | .  .  . | .  .  . | .  .  . | .  .  . | .  .  . | .  .  . |

Age, Sex, Marital Status, Income, Social Support Availability

General Health, Mental Health, Healthy Aging, Life Satisfaction, BMI

ER Use, Hospital Visits, Nursing Home

Caregiving, Puzzles, Music, Community Activities, Social Media

1. Sociodemographic

2. General Health

3. Healthcare Utilization

4. Non-Health Behaviours

**Step 5:** Characterize clusters based of summary statistics of clusters (e.g., cluster 1 defined by higher than average sedentary behaviour, walking, smoking, and alcohol consumption and lower exercise)
