# Supplementary material for: Clustering of Unhealthy Behaviors: Protocol for a Multiple Behavior Analysis of Data From the Canadian Longitudinal Study on Aging
Source: JMIR Res Protoc. 2021 Jun 11;10(6):e24887. doi: 10.2196/24887 (PMC8235290; doi:10.2196/24887)
Supplement: Multimedia Appendix 3 [file resprot_v10i6e24887_app3.docx]

**Multimedia Appendix 3**

Graphical representation of the network analysis and community detection process based on analysis on simulated data (n = 1000).


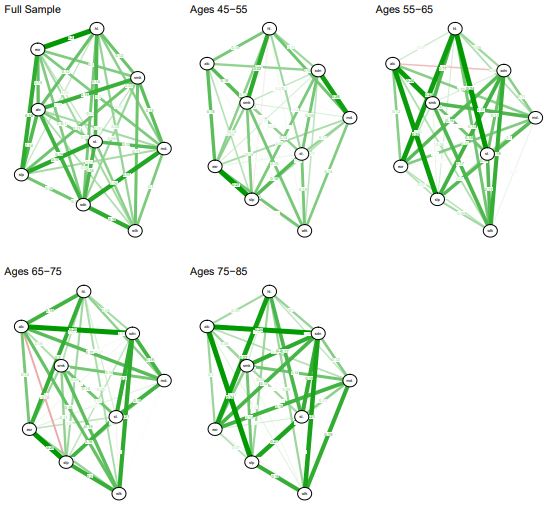


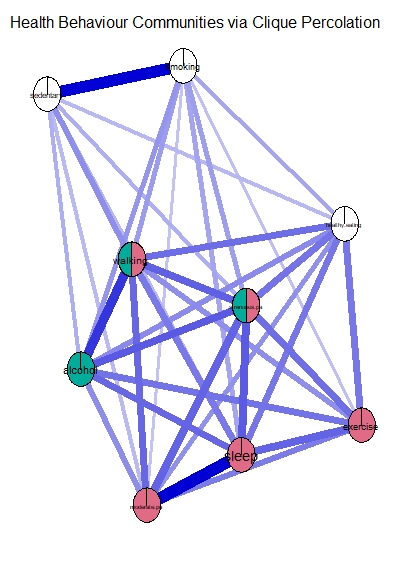

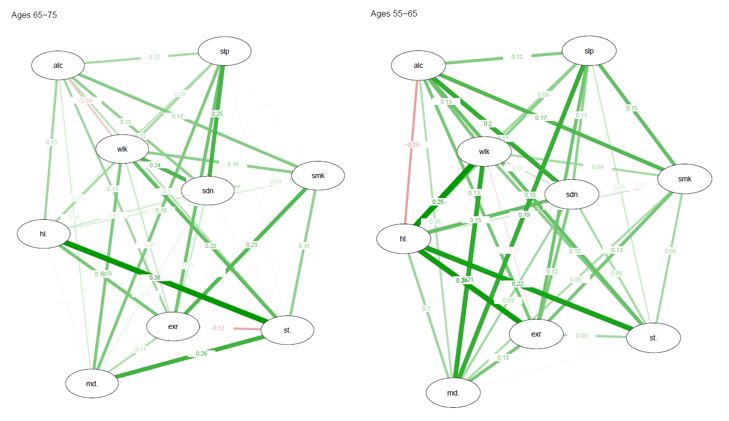


**Step 3:** Perform network comparisons tests to compare networks between age groups.

**Step 4:** Use the clique percolation algorithm for community detection to detect overlapping communities of health behaviours

**Step 1 & 2:** Compute and visualize networks for the full sample and for each age group.
