# Supplementary material for: Clustering of Unhealthy Behaviors: Protocol for a Multiple Behavior Analysis of Data From the Canadian Longitudinal Study on Aging
Source: JMIR Res Protoc. 2021 Jun 11;10(6):e24887. doi: 10.2196/24887 (PMC8235290; doi:10.2196/24887)
Supplement: Multimedia Appendix 4 [file resprot_v10i6e24887_app4.pdf]

Review Type/Type d'évaluation: Committee Member 1/Membre de comité 1
Name of Applicant/Nom du chercheur: Presseau, Justin
Application No./Numéro de demande: 408848
Agency/Agence: CIHR/IRSC
Competition/Concours: 2018-09-05 Catalyst Grant: Analysis of Canadian Longitudinal Study in Aging (CLSA) Data/Subvention catalyseur: Analyse des données de ÉCLV
Committee/Comité: Catalyst Grant: Analysis of CLSA Data/Subvention catalyseur : Analyse des données de l'ÉLCV
Title/Titre: Clustering of (un)healthy behaviours in older Canadians

---

Assessment/Évaluation:

This application seeks to undertake a pretty straightforward analysis to examine how health behaviours cluster in the CLSA and to describe the characteristics of people in different clusters, their health and life satisfaction and health care utilisation. It seems highly likely that this analysis will be possible and will produce descriptive results. What is very unclear is whether it will produce anything that the applicants don't know already and whether it will produce insights that impact on preventive strategies. The applicants say that the study will" inform the development of multiple interventions tailored to sub-populations of older adults defined by the behaviours that cluster within them". It is not clear that this is very likely nor whether "targeted interventions to support healthy aging" will result in better outcomes than general approaches to helping to enhance health related behaviours in all individuals.

Review Type/Type d'évaluation: Committee Member 2/Membre de comité 2
Name of Applicant/Nom du chercheur: Presseau, Justin
Application No./Numéro de demande: 408848
Agency/Agence: CIHR/IRSC
Competition/Concours: 2018-09-05 Catalyst Grant: Analysis of Canadian Longitudinal Study in Aging (CLSA) Data/Subvention catalyseur: Analyse des données de ÉCLV
Committee/Comité: Catalyst Grant: Analysis of CLSA Data/Subvention catalyseur : Analyse des données de l'ÉLCV
Title/Titre: Clustering of (un)healthy behaviours in older Canadians

---

Assessment/Évaluation:

Innovative clusters show associations and frequency of the behaviours. Limit is the cross-sectional nature of the data. Clustering by age or gender does not allow any signal of the time frame (length) of those behaviors or of the stability of the inference. The variables used do indicate frequency which allows this work to be conducted.

Team is an interesting group of excellent experienced researchers but that experience is for other datasets. While certainly relevant and impressive experience of longitudinal data is presented, the one-year time frame given for the work makes the ability to quickly use the CLSA data less likely. The strength of the team assembled is its interdisciplinary nature. The group is clearly well positioned in Ottawa to use the data and work with local officlals which is important for the knowledge translation and dissemination. Another strength is the willingness of the mid-career PI (presseau) to use the data and be well positioned to continue to explore these relationships over future waves.

Review Type/Type d'évaluation: Committee Member 3/Membre de comité 3
Name of Applicant/Nom du chercheur: Presseau, Justin
Application No./Numéro de demande: 408848
Agency/Agence: CIHR/IRSC
Competition/Concours: 2018-09-05 Catalyst Grant: Analysis of Canadian Longitudinal Study in Aging (CLSA) Data/Subvention catalyseur: Analyse des données de ÉCLV
Committee/Comité: Catalyst Grant: Analysis of CLSA Data/Subvention catalyseur : Analyse des données de l'ÉLCV
Title/Titre: Clustering of (un)healthy behaviours in older Canadians

---

Assessment/Évaluation:

The writing of the proposal is excellent. The project aims to study how an individual's behavior affects the behavior of other individuals and how these patterns are associated to health and life satisfaction, taking into account sociodemographic aspects. It is a nice project.
The application of the proposed methodology to Canadian data is original. Similar analysis with interesting results were done in other countries, like the UK, but this analysis has never been done for Canada. The applicants propose to analyze how the health behaviours cluster by age, sex. It could also be interesting to consider other dimensions, like provinces or income.
The team and the environment are excellent.
